# Effect of Cardiac Rehabilitation Program on Exercise Capacity in Women Undergoing Coronary Artery Bypass Graft in Hamadan-Iran

**Published:** 2010

**Authors:** Ramin Shabani, Abas A. Gaeini, Mohamad R. Nikoo, Hojatollah Nikbackt, Majid Sadegifar

**Affiliations:** 1 Exercise Sciences Department, Science & Research Branch, Islamic Azad University, Tehran, Iran; 2 Exercise Sciences Department, Tehran University, Tehran, Iran; 3 University of Welfare and Rehabilitation Sciences, Tehran, Iran; 4 Exercise Sciences Department, Science and Research Branch, Islamic Azad University, Tehran, Iran; 5 Boali Sina University, Hamadan, Iran

**Keywords:** Cardiac Rehabilitation, Women’s Health, Treadmill Test, Rate Pressure Product, Walk Test

## Abstract

**Objectives:**

The purpose of this study was to determine the effects of cardiac rehabilitation program (CRP) on exercise capacity and rate pressure product (RPP) in Iranian female patients undergoing coronary artery bypass grafting (CABG) in Hamadan, Iran.

**Methods:**

Sixty women after CABG were assigned into an exercise group (n = 30, mean age 58.5 ± 10.8 years), who performed physical training for 12 weeks, or a control group (n = 30, mean age 59.3 ± 8.6 years) who received usual care. Functional capacity and RPP were evaluated by six minute walking test (6MWT) and exercise test.

**Results:**

In comparison to before training, significant increases of estimated exercise capacity (10.72 ± 1.30 vs. 7.72 ± 1.6 MET’s, respectively) as well as 6MWT (556 ± 66.1 vs. 375.2 ± 28.1 meters, respectively) were observed in exercise group after 12 weeks training (P<0.001). Women increased their exercise duration time (464.6 ± 107.3 vs. 311.2 ± 101.7 seconds, respectively) by 49.2% and RPP (22361 ± 3206 vs. 20270 ± 2704.1, respectively) by 10.3% after training (P<0.001). However, no significant differences were found before and after CRP in the control group.

**Conclusions:**

Women referred for rehabilitation have similar levels of compliance and improvement in exercise capacity and supply of oxygen to cardiac muscles (measured by peak myocardial oxygen consumption). After CRP, women demonstrated significant improvements in exercise duration time, 6MWT, RPP and supply of oxygen to cardiac muscles. CRP can play an important role in improving functional independence in women.

## INTRODUCTION

Cardiovascular disease is the leading cause of death in Iran, which accounts for 46% of overall mortality.^1^ Cardiac rehabilitation program (CRP) has been acknowledged to be an important component in the management of patients with coronary heart disease,^2^ and exercise training (ET) has been a core component of CRP with the assumption that enhanced physical capacity will lead to improved daily physical activity,^3^,^4^ in turn favorably influencing an individual’s lifestyle,^5^ and thus reducing the likelihood of further cardiac events.^6^,^7^ The value of ET has been widely recognized in the literature; ET reduces mortality in patients with coronary artery disease^8^ and the long-term prognosis of patient with known coronary artery disease is strongly related to exercise capacity. Previous studies have revealed that ET in men with cardiac diseases induces a significant increase in exercise capacity.^9^,^10^ However, there are a few published data on the effect of CRP in women exercise capacity.^11^ In addition, older age, lower exercise levels and reduced functional capacity or co-morbid conditions are barriers to physical activities in women with ischaemic heart disease.^12^ On the other hands, rate pressure product (RPP) is a major determinant of cardiac oxygen consumption. It is an important indicator of ventricular function. RPP varies with exercise. The peak rate pressure product (PRPP) which gives an accurate reflection of the myocardial oxygen demand and myocardial workload is the RPP at peak of exercise. The higher the PRPP, the more will be myocardial oxygen consumption (MVO_2_).^13^ MVO_2_ is a good indicator of the response of the coronary circulation to increased myocardial oxygen demand.^14^ The ability to reach higher PRPP is associated with more adequate coronary perfusion. Thus, the low value of PRPP suggests significant compromise of coronary perfusion and decreased left ventricular function.^12^ The current study attempted to determine the effects of CRP on exercise capacity, and RPP, an index of myocardial oxygen consumption in Iranian women patients undergoing CABG.

## METHODS

### 

#### Patients

We conducted a quasi-experimental and perspective design study with a non-randomized, pretest-posttest control group. Sixty subjects were allocated to either a CRP group (n= 30, mean age of 58.5 ± 10.8 years) or a control group (n=30, mean age of 59.3 ± 8.6 years). At baseline and after 12 weeks, exercise capacity was measured. Patients who were not able to participate in the CRP because of distance or needing to work were assigned to the control group, after matching on age and left ventricular ejection fraction (LVEF) to the subject previously enrolled to the CRP group. All patients had a recent (<2 months) CABG and no contraindication to undergo a cardiac rehabilitation program. Inclusion criteria were as follows: resting LVEF≥30%, no complications during hospitalization, stable medication regimen, and study physician’s approval. All patients performed exercise tolerance tests before and after CRP. The study was approved by the vicedean for research and research Ethics Committee of the Hamedan University of Medical Sciences, and all participants were given written informed consent. Prior to enrollment into the study, the participants were required to have a medical examination, and questionnaires regarding their anthropometric data, medical history and physical activity were completed.

#### Data Collection

Functional capacity was measured by 6MWT and exercise test (ET). In this regard, 6MWT was done at admission and after CRP. ET was prepared on a treadmill using standard Bruce protocol^15^ before and after CRP. During the test, patients were encouraged to continue exercising until they were limited by symptoms. The cardiac rehabilitation program comprised 36 exercise sessions, scheduled over 12 weeks.

#### Exercise Protocol

After concluding the baseline tests, the patients in exercise group were enrolled in a supervised exercise and rehabilitation program, which included stretching and warm-up exercise (10-15 min), endurance training (15-20 min), resistance training (10-15 min) and cool-down/relaxation exercise (10 min). Exercise intensity began at 40% to 50% of maximum heart rate reserve or as symptoms allowed and gradually progressed to 60% to 80% of maxi-mum heart rate reserve. Resistance training was recommended 3 days per week and consisted of 8 to 10 exercises covering the major muscle groups in chest, shoulders, arms, back, abdomen, thigh and lower legs. Resistance (weight) was set at 30% to 40% of 1 repetition maximum for upper body and 50% to 60% for lower-body exercises. When 12 to 15 repetitions could be accomplished with little difficulty, the weight was increased. Furthermore, by using moderate weight and gradually increasing the workload in stages, there was less risk of musculoskeletal injury while maintaining effectiveness of the workout.^16^ Having passed the second week of the program, the patients were encouraged to do daily walking exercises for at least 45 minutes subsequent to the warm-up exercises for 1-3 times a week.

#### Statistical Analysis

Using SPSS statistical package, version 17, the data were analyzed. The results were given as mean ± SD for continuous variables and the mean scores before and after cardiac rehabilitation were compared using paired t-test. Statistical significance was set at P<0.05.

## RESULTS

All participants safely completed a 12-week training phase, with no significant adverse effects. Medication was continued during testing. In both groups, LVEF was decreased. Baseline clinical features and demographic characteristics of patients are showed in Table 1. The mean ages and body mass index (BMI) of the patients in each group were similar (P>0.05).

**Table 1:** Baseline clinical features and demographic characteristics of study participants

	Exercise group (n=30)	Control group (n=30)
Weight (kg)	66.1 (19.5)	67.7(20.2)
BMI (kg m^-2^)	27.4(6.8)	25.8(5.4)
METs	7.72(1.60)	8.1(1.92)
EF,%	49(8)	50(7)
BP Rest(mm Hg)	131/79(14/6)	128/89(12/8)
Tobacco use, n (%)	2(6%)	1(3%)
Hypertension, n (%)	12(40%)	13(43%)
Diabetes mellitus, n (%)	8(26%)	9(30%)
Dyslipidemia, n (%)	14(46%)	11(36%)

BMI: body mass index, BP: blood pressure, EF: ejection fraction.

Data present mean (SD)

Mean left ventricular ejection fraction (LVEF) was not significantly different in the exercise compared to control group (49 ± 8% vs. 50 ± 8%, P = 0.08). Medication profiles are listed in table 2.

**Table 2:** Medications used by participants

	Exercise group (n=30)	Control group (n=30)
β blocker, n(%)	23(76%)	24(80%)
Nitrate, n (%)	18(60%)	20(66%)
Digoxin, n (%)	25(83%)	23(76%)
Anticoagulant, n (%)	30(100%)	30(100%)
ACE inhibitors, n (%)	12(40%)	13(43%)
Statins, n (%)	14(46%)	11(36%)

ACE: Angiotensin-converting enzyme

Exercise capacity was measured using metabolic equivalent (METs) and exercise time, and physical capacity was assessed using 6-minute walk test (6MWT). Exercise time and 6MWT did not differ between the two groups at study entry (P>0.05). Functional parameters improved significantly in the exercise compared to the control group after the training (Table 3). Compared to the baseline, after 12 weeks of training, significant increases of exercise duration time as well as 6MWT was observed in the exercise group: 311.2 ± 101.7 vs. 464.6 ± 107.3 seconds and 375.2 ± 28.1 vs. 556 ± 66.1 meters (P<0.001), respectively. On the other hands, compared to the baseline, maximum systolic blood pressure as well as maximum heart rate was observed in the exercise group after intervention: 151.5 ± 18.7 vs. 155 ± 16.8 mmHg (P=0.008) and 135.6 ± 23.5 vs. 144.2 ± 20.8 beat/min (P=0.004), respectively and RPP increased from 20270 ± 2704.1 to 22361 ± 3206 (P<0.001) during exercise test. After training sessions, significant changes were not found in exercise test duration, maximum systolic blood pressure, maximum heart rate, and rate pressure product during exercise test in the control group (P>0.05).

**Table 3:** Comparison of exercise capacity variables before and after the training in women Mean (SD)

	Exercise group(n=30)		Control group (n=30)		p value	p value
	I:Baseline	II: 3 months	III: Baseline	IV: 3 months	I vs. II	III vs. IV
Exercise time (Second) (Bruce protocol)	311.2(101.7)	464.6(107.3)	317(98)	329(105)	<0.001*	NS
Max sys BP (mmHg)	151.5(18.7)	155(16.8)	148(14.2)	150(12.9)	0.008 *	NS
Max heart rate (beat/min)	135.6(23.5)	144.2(20.8)	130.1(15.8)	132(17.2)	0.004*	NS
METs	7.72(1.6)	10.72(1.30)	7.27(1.95)	7.50(2.02)	<0.001*	NS
RPP	20270(2704.1)	22361(3206)	19240(2580)	19908(2750)	<0.001*	NS
6MWT, m	375.2(28.1)	556(66.1)	383(32)	390(39)	<0.001*	NS

METs: metabolic equivalent, Max sys BP: maximum systolic blood pressure, RPP: rate pressure product

## DISCUSSION

Findings of this study indicated that a supervised hospital-based physical training (endurance and resistance exercise) is relatively safe. In our study, exercise capacity improved significantly in the exercise compared to the control group.

### 

#### Exercise Capacity after 12-Week CRP

Although patients from both groups had similar reduced baseline exercise and physical capacity, CRP significantly increased exercise capacity, assessed by exercise duration time and METs (during exercise test). Women increased their exercise duration time by 49.2% and METs by 39.1%. Cannistra et al.^11^ showed that women participating in a cardiac rehabilitation exercise program for 12 weeks increased their peak METs by 30% compared with a 21% increase in men. Women had similar rates of compliance and achieved the same improvement in exercise capacity with training which was consistent with previous studies.^17^–^20^

#### 6MWT after 12- Week CRP

In the current study, although patients from both groups had similar reduced baseline exercise tolerance, a supervised, relatively short-term (12-week) hospital based exercise and rehabilitation program, significantly increased physical capacity, assessed by a longer walking distance (a 6-minute walk test). Fiorina et al. showed that 6MWT was well tolerated in all patients. The mean distance walked in 1370 patients was 304 ± 89 m (corresponding to 58 ± 15% of the predicted value). Distances walked were significantly shorter in older patients than in younger ones (P<0.05) and in women compared to men (251 ± 78 m and 53 ± 15%, vs. 328 ± 34 m and 60 ± 14%, P<0.001) and in no CABG compared to CABG patients (285 ± 91 m vs. 303 ± 84 m, P<0.001. In the subgroup of patients repeating the 6MWT after the rehabilitation program, the distance walked significantly increased (from 281 ± 90 m and 51 ± 76% vs. 411 ± 107 m and 77 ± 81%, P<0.001).^21^

#### RPP after 12-Week CRP

Cardiac rehabilitation programs should focus on enhancing RPP max.^22^ Compared to before training, in exercise group after training, maximum heart rate (135.6 ± 23.5 vs. 144.2 ± 20.8 beats/min, respectively), maximum systolic BP (151.5 ± 18.7 vs. 155 ± 16.8 mmHg, respectively), and RPP (20270 ± 2704.1 vs. 22361 ± 3206 beats·min ^−1^·mmHg, respectively) significantly increased. This study revealed that RPP (an easy, reliable, and indirect measure of peak myocardial oxygen consumption) increased at maximum effort in exercise test by 10.3% (P<0.001). Many study reports have suggested the necessity of exercise training which improves PRPP in patients with cardiac disease. These findings are similar to those described by May et al.^23^ and others.^24^

It should be noted that the exercise modality in the present study, a moderate-intensity resistance training program with aerobic exercise, was well-tolerated by closely supervised and monitored patients and resulted in no adverse events. The resistance modality was probably associated with favorable responses. Vincent et al.^25^ showed that resistance exercise should be incorporated into a comprehensive exercise regimen to increase muscular strength, cardiorespiratory endurance, and physical function.

This study pointed out that women referred for rehabilitation have similar levels of compliance and improvement in exercise capacity and supply of oxygen to cardiac muscles (measured by peak myocardial oxygen consumption). Many study reports have suggested the necessity of exercise training which improves exercise capacity in women patients after coronary artery disease.^26^,^27^ These results may remind physicians that women as well as men can potentially benefit from cardiac rehabilitation programs. However, more knowledge is needed about the response of women to physical training.

#### Limitation

Our conclusions are based on relatively young women (<70 years) with stable CHD who were referred for cardiac rehabilitation, and may not apply across the spectrum.
